# Multi-level Hierarchical Complex Behavior Monitoring System for Dog Psychological Separation Anxiety Symptoms

**DOI:** 10.3390/s22041556

**Published:** 2022-02-17

**Authors:** Huasang Wang, Othmane Atif, Jirong Tian, Jonguk Lee, Daihee Park, Yongwha Chung

**Affiliations:** 1 Department of Computer and Information Science, Sejong Campus, Korea University, Sejong City 30019, Korea; huasangwang@korea.ac.kr (H.W.); osuman@korea.ac.kr (O.A.); 2 Department of Animal Science, University of California, Davis, CA 95616, USA; jirtian@ucdavis.edu; 3 Department of Computer Convergence Software, Sejong Campus, Korea University, Sejong City 30019, Korea; ychungy@korea.ac.kr

**Keywords:** complex event processing (CEP), long short-term memory (LSTM), fuzzy logic, pattern recognition, separation anxiety, animal–computer interaction (ACI), sensor

## Abstract

An increasing number of people own dogs due to the emotional benefits they bring to their owners. However, many owners are forced to leave their dogs at home alone, increasing the risk of developing psychological disorders such as separation anxiety, typically accompanied by complex behavioral symptoms including excessive vocalization and destructive behavior. Hence, this work proposes a multi-level hierarchical early detection system for psychological Separation Anxiety (SA) symptoms detection that automatically monitors home-alone dogs starting from the most fundamental postures, followed by atomic behaviors, and then detecting separation anxiety-related complex behaviors. Stacked Long Short-Term Memory (LSTM) is utilized at the lowest level to recognize postures using time-series data from wearable sensors. Then, the recognized postures are input into a Complex Event Processing (CEP) engine that relies on knowledge rules employing fuzzy logic (Fuzzy-CEP) for atomic behaviors level and higher complex behaviors level identification. The proposed method is evaluated utilizing data collected from eight dogs recruited based on clinical inclusion criteria. The experimental results show that our system achieves approximately an F1-score of 0.86, proving its efficiency in separation anxiety symptomatic complex behavior monitoring of a home-alone dog.

## 1. Introduction

The number of dogs raised as pets increased due to the beneficial impacts on the owners’ mental health, which is more evident for owners living alone or with fewer family members [1,2,3]. Unfortunately, despite owners being emotionally attached to their dogs, it is practically unfeasible for them to constantly look after their dogs. Thus, owners are forced to leave them at home alone in some cases, increasing the risks of dogs developing psychological disorders such as Separation Anxiety (SA) [4,5]. The latter is considered the most common dog psychiatric disorder, often accompanied by complex behavioral symptoms, such as high-frequency destructive behavior, which damages their surrounding environment, e.g., furniture and appliances, and excessive vocalization, which disturbs the neighboring community [6,7,8]. In addition, these undesired complex behavioral symptoms are the primary reasons forcing owners to relinquish their dogs [9,10]. In America alone, nearly 670,000 dogs are euthanized each year, mainly due to behavioral problems related to psychiatric disorders [11]. Therefore, to improve dogs’ welfare and prevent them from developing separation anxiety, it is necessary to observe and monitor abnormal complex behavioral symptoms in advance and treat them successfully [10]. However, since SA is only triggered by the owner’s real or perceived absence [12], direct observation revealing the dog’s behavioral symptoms can be disruptive. In the past 20 years, SA symptom observation in dogs was already studied utilizing subjective ratings such as interviewing the owners [6,12] or relying on manual behavior recognition from recorded videos [4,6]. Nevertheless, these methods are laborious and inefficient, and they cannot automatically detect the early psychological symptoms of SA.

Spurred by the deficiencies of current methods, this study aims to propose a novel approach based on computer techniques, instead of manual methods, to automatically monitor a cage-free dog’s early primary SA symptomatic complex behaviors identified as ‘Excessive destructive behavior’, ‘Excessive exploratory behavior’, and ‘Excessive vocalization’. The current recording-based manual observation scheme first observes the dog’s head and body postures (poses and motions) to identify the atomic behaviors and then aggregate the latter into complex behaviors [6,8,13,14,15]. By summarizing these observation methods, we created a taxonomy of the dog activities involving the three levels presented in Table 1. Level-1 activities represent a dog’s body pose or motion at a specific time [16]. In this case, a set of head or body postures compose an atomic behavior. For example, the ‘Walking’ behavior comprises a set of ‘Walk’ postures. Accordingly, the Level-2 activities represent the dog’s atomic behavior, a fundamental behavior [4,17]. Finally, the Level-3 activities represent the dog’s abnormal complex behavior, divisible and aggregated by a set of high-frequency atomic behaviors [4,8]. For instance, the complex abnormal behavior of ‘Excessive vocalization’ is aggregated by a set of high-frequency ‘Barking’ atomic behaviors [8,10,18].

Various studies aiming to recognize and detect dog activities at different levels were proposed, with Table 2 summarizing the most important ones [17,19,20,21,22,23,24,25,26,27,28,29,30,31,32,33]. Early research focused on Level-1 dog posture recognition, leading to the initial and essential work towards dog behavior recognition. Recent approaches focused on Level-2 dog behavior recognition and started to detect abnormal behaviors related to a dog’s welfare. Despite the research proposing several methods, only a few recent studies managed a Level-2 atomic behavior recognition accuracy of 90% [25,27,31]. However, recent studies are unable to detect separation anxiety symptomatic behaviors for the following limitations:

1. Although some studies included potentially abnormal behaviors relevant to a dog’s well-being, they mainly focused on Level-1 postures or Level-2 abnormal atomic behaviors, e.g., ‘Barking’. Nevertheless, these techniques are insufficient to determine the specific disorder that dogs might suffer from. For instance, the atomic behavior of barking can be related to noise phobia and be triggered when the dog hears outside noise, and the behavior is only considered a separation anxiety-related abnormal behavior when its frequency is high. Hence, solely recognizing the potential abnormal atomic behavior cannot be directly used to provide an accurate diagnosis of separation anxiety symptoms.
2. To the best of our knowledge, only one separation anxiety reduction system [34] includes Level-3 separation anxiety-related symptomatic complex behaviors. However, training this system requires the owner’s direct participation, e.g., the owner labels the complex behaviors, such as ‘Destructive behavior’, using a smartphone. Hence, this architecture is unable to monitor complex behavioral symptom scenarios automatically.
3. The feasibility of implementing a dog automatic monitoring system related to psychological separation anxiety symptoms has not been reported yet. Thus, the current research gap increases the challenge of automatically inferring Level-3 complex behaviors from lower levels [35].

To address these limitations, this paper proposes an end-to-end, knowledge-based multi-level hierarchical system, which automatically monitors a home-alone cage-free dog starting from Level-1 (head and body postures), going through Level-2 (atomic behaviors), and reaching Level-3 (separation anxiety-related symptomatic complex behaviors). At the lowest level, we apply stacked Long Short-Term Memory (LSTM) models to recognize the dog’s posture using preprocessed time-series data collected from head and body wearable sensors. The stacked LSTM guarantees effective and stable performance for posture recognition when using time-series data [36,37]. Then, based on the extracted head and body postures, we design a dog behaviors detection algorithm using Complex Event Processing (CEP) with dog behavior knowledge-based pattern rules for Level-2 atomic behaviors and Level-3 complex behaviors identification. The suggested CEP technology models the knowledge hierarchy and automatically detects meaningful complex events [38,39]. However, it is challenging to define ambiguous and uncertain psychological knowledge using CEP rules. For instance, ‘Excessive vocalization’ cannot be quantified to build CEP numerical pattern rules. To overcome the limitations of the basic CEP rules, we introduce fuzzy logic that handles imprecision and effectively represents psychological knowledge [40,41], extending the basic CEP rules for Level-3 symptomatic complex behavior monitoring. To evaluate the proposed system, we develop a prototype system with real-world datasets that include eight dogs’ daily routines and separation anxiety scenarios. The dogs were recruited based on clinic separation anxiety inclusion criteria [42].

The remainder of this paper is structured as follows: Section 2 describes the proposed method for automatically monitoring a freely moving dog’s separation anxiety symptomatic complex behavior; Section 3 presents the experimental setup and analyzes the results; finally, Section 4 concludes this paper.

## 2. Proposed Method

### 2.1. System Structure

**Figure 1:**
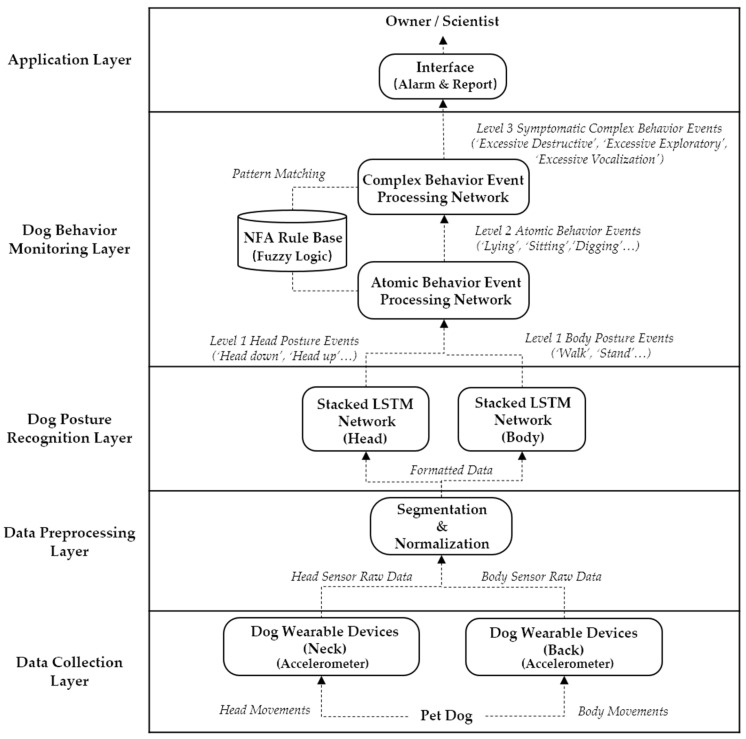
Proposed dog monitoring system architecture detects separation anxiety symptomatic complex behaviors and primarily focuses on ‘Excessive destructive behavior’, ‘Excessive exploratory behaviors’, and ‘Excessive vocalization’.

The proposed dog monitoring system architecture is illustrated in Figure 1, comprising five layers: data collection, data preprocessing, dog posture recognition, dog behavior monitoring, and application layer.

### 2.2. Data Collection Layer and Data Preprocessing Layer

As mentioned in Section 1, the Level-1 posture analysis is the first crucial step towards understanding dog behavior and detecting a dog’s symptomatic complex behavior related to separation anxiety [25]. Moreover, the symptomatic complex behaviors are not independent of each other. For example, in most cases, dogs that present head posture-related complex behavior ‘Excessive vocalization’, exhibit body posture-related complex behavior ‘Excessive destructive behavior’ simultaneously. Therefore, it is necessary to collect head and body postures in dogs concurrently when monitoring their behavior. In this work, the data collection layer relies on wearable devices with tri-axial accelerometers to automatically collect a freely moving dog’s head and the body posture raw time-series data. The wearable sensor is convenient for detecting postures and is practical for real-world situations as it does not require well-controlled environments [43]. Furthermore, accelerometers already proved their measurement abilities for the pose and motion of a wide range of species [25]. In prior works, head and body posture data collection explored various sensor locations on a dog’s body. It concluded that back-mounted and neck-mounted devices produced high-quality data for the head and body posture estimation [25,44], achieving a recognition accuracy of approximately 90% [25,27,30]. Therefore, we use two dog wearable devices with tri-axial accelerometers on the dog’s neck and back to collect the dog’s head and body posture raw data, respectively.

The data preprocessing layer aims to dynamically segment, normalize, and format the raw time-series data as an input stream to the LSTM-based model, guaranteeing continuous dog posture recognition. Traditionally, sensor-based data segmentation method uses sliding windows to detect the activity’s start and end time [45]. In this work, we set the sliding window size as one second to ensure the system detects each posture’s central part. Besides, due to the various dog sizes, the accelerometer data have different value ranges, with larger values dominating the LSTM network training and ultimately imposing a natural bias [46]. Therefore, the min–max normalization method is utilized to normalize the time-series data between the values 0 and 1 [47]. After normalization, the data are converted into an appropriate format and input to the LSTM network as a 3D vector with the shape (Samples) × (Timesteps) × (Features). In this study, ‘Timesteps’ is set to the value 50, which corresponds to the sequence of data received from the sensor during one second, and the ‘Features’ are the accelerometer’s *x*-, *y*-, and *z*-axis.

### 2.3. Dog Posture Recognition Layer

The purpose of this layer is to classify the preprocessed dog’s motion data into an understandable head or body posture category. These postures are the hierarchy’s basic level (Level 1) activities that are used to detect the higher-level atomic and complex behaviors. For posture recognition, earlier methods using models such as SVM relied on hand-crafted features extracted from the input data through fixed mathematical rules [21,22,46]. However, to engineer hand-crafted features, domain knowledge about the specific application is required [48]. Recently, deep learning techniques were widely employed in several recognition fields, automating the feature extraction process without requiring domain knowledge [49,50,51]. One of the deep learning models, the stacked LSTMs, is a well-suited network for recognizing sensor time-series data and enhancing the model’s accuracy [36,37,46,52,53]. Therefore, we employ stacked LSTMs as our system base analyzer to classify the Level-1 dog’s postures and motions.

**Figure 2:**
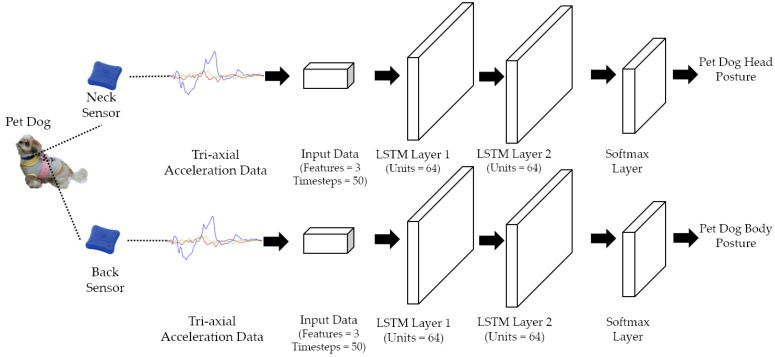
Structure of two parallel stacked Long Short-Term Memory (LSTM) networks for dog head and body posture recognition.

The proposed stacked LSTM network structure for dog posture recognition is illustrated in Figure 2, where the two stacked LSTM networks for dog head and body posture recognition are independent and parallel. Each stacked LSTM network comprises two LSTM layers of 64 units each. The output layer is a softmax layer calculating the probability and classifying the data into one of the Level-1 postures presented in Table 1. The head and body postures are then forwarded to the next layer to be used to detect higher level behaviors.

### 2.4. Dog Behavior Monitoring Layer

This layer abstracts a multi-level knowledge-based hierarchical structure from the previous manual recognition methods to recognize Level-2 and Level-3 dog behaviors effectively. Based on the hierarchical structure, we utilize the Complex Event Processing (CEP) technology with knowledge rules to automatically detect Level-2 and Level-3 dog behaviors. The CEP combined with knowledge-based rules can automatically identify causality patterns and detect meaningful complex events with time relationships. However, while basic CEP rules are enough to detect level 2 atomic behaviors based on level 1 postures, it is challenging to employ them to effectively express the Level-3 psychological separation anxiety-related behavioral information based on specific indicators. For example, it is difficult to quantify the number of ‘Barking’ to detect the abnormal status ‘Excessive vocalization’. Therefore, we introduce a fuzzy logic concept to extend the effectiveness of the CEP rules, constituting a Fuzzy-CEP dog behavior monitoring system.

#### 2.4.1. Abstraction Hierarchy of Dog Separation Anxiety-Related Behaviors

**Figure 3:**
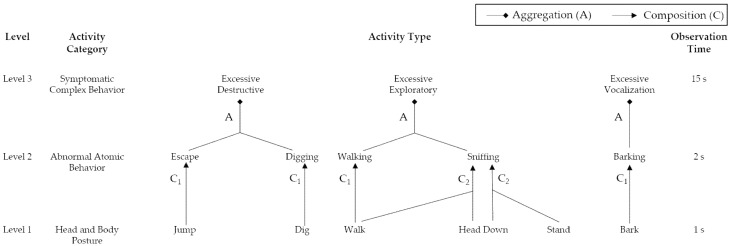
Abstraction hierarchy for dog separation anxiety-related complex behaviors detection.

As previously explained, existing manual observation methods of dog behavior highlighted that a set of understandable primitive postures compose an atomic behavior [13,15]. Additionally, a set of temporal and coherent atomic behaviors can be aggregated into a dog’s symptomatic complex behavior [8]. Therefore, we exploit the hierarchy concept, an effective method for expressing the aggregation or composition relationship between activities [43,54]. Figure 3 illustrates the proposed three-level hierarchy for primary complex behavioral symptoms appropriate for separation anxiety detection. As a reminder, the three levels defined in Table 1 include Level-1 (head and body postures), Level-2 (atomic behaviors), and Level-3 (separation anxiety-related symptomatic complex behaviors). Composition C_1_ and C_2_ are the two types of relationships between Level-1 and Level-2. The C_1_ relationship represents that an atomic behavior comprises a set of identical postures during the observation time, while the C_2_ relationship denotes that atomic behavior comprises various postures during the observation time. For instance, atomic behavior ‘Sniffing’ involves body posture ‘Walk’ and head posture ‘Head down’. The Aggregation (A) is the relationship between Level-2 and Level-3, representing complex behaviors aggregated by related lower-level atomic behaviors that are more frequent than when the owner is at home [13]. For instance, the complex behavior ‘Excessive exploratory behavior’ is aggregated by excessive and higher-frequency atomic behavior ‘Walking’ and atomic behavior ‘Sniffing’.

#### 2.4.2. Hierarchy Modeling for Dog Behavior Automatic Detection

The basic approach involves using the rule-based method to automatically monitor a dog’s behavior based on the hierarchy presented in Figure 3 [55,56]. However, the latter method is limited in using simple rules for dog behavior detection, and thus rule-based techniques cannot infer the higher-level activity from a set of lower-level activities with time relationships [57,58]. In this work, we exploit the complex event processing technology as our primary method to address this issue. The CEP can simultaneously and automatically identify meaningful events and generate higher-level events based on relationships, i.e., time and aggregation relationships [59,60]. Furthermore, CEP rules can be extended by custom aggregate functions according to fundamental requirements [61].

**Figure 4:**
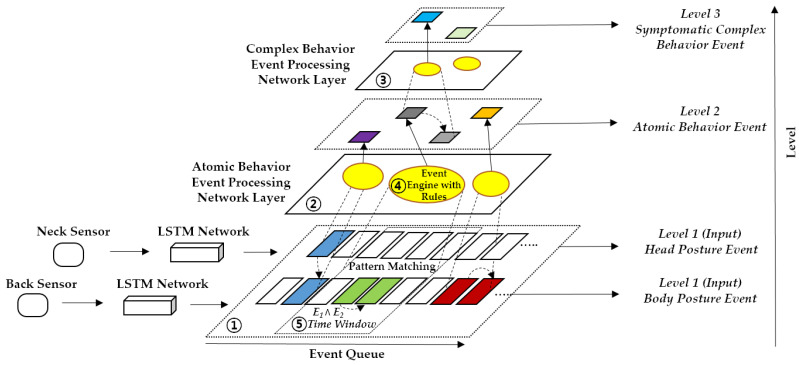
Complex Event Processing (CEP) hierarchy structure for dog behavior monitoring.

Figure 4 shows how the CEP technology models a dog’s behavior based on hierarchy: (1) the Level-1 head and body postures are input into event streams of the CEP hierarchy, calculated by stacked LSTM networks that utilize the preprocessed sensor datasets; (2) the Level-2 dog atomic behaviors are detected through the atomic behavior Event Processing Network (EPN) that relies on the extracted postures; (3) the Level-3 complex behaviors are then detected by the complex behavior EPN that exploits the related atomic behaviors. Each network includes the event processing engines and the CEP rules; (4) the CEP engine selects the lower-level events that satisfy the CEP pattern rules and generates higher-level events; (5) the CEP rule is defined by events and event constructors, expressing the relationships between the events [41]. In Figure 4, colored events represent the ones that compose a pattern when a matching has occurred within the time window frame. Accordingly, events using the same color correspond to a detected pattern. In this work, the events are the activities of each layer modeled as presented next. Specifically, a posture event (P) is denoted as:

(1) P=E (id,s, p, t),

where id is the dog’s subject ID, s is the sensor ID, p is the dog’s posture class, and t is the posture’s timestamp. An atomic behavior event (A) is denoted as:

(2) $$A=E\;\left(id,\;ab,\;t_{s},t_{e}\right),\;\;t_{s}<t_{e},$$

where ab is the dog’s atomic behavior class, $t_{s},\;t_{e}$ are the behavior event starting time and ending time.$\;t_{e}-t_{s}$ is observation time interval. A complex behavior event (C) is denoted as:

(3) $$C=E\;\left(id,cb,\;d,t_{s},t_{e}\right),\;\;t_{s}<t_{e},$$

where cb is the dog’s complex behavior class, and d is the symptom state of complex behavior.

This work adapts the CEP event constructors for dog behavior detection presented in Table 3. Additionally, we create a fuzzy function appropriate for the dog’s psychological separation anxiety symptomatic complex behavior detection.

The specific dog behavior detection rules are introduced in Table 4, which model the C_1_ and C_2_ relationship combination of the atomic behaviors in the atomic behavior event processing network. For instance, if the dog maintains posture ‘Dig’ without changing within two seconds of observation, the atomic behavior ‘Digging’ will be generated. Besides, if the body posture ‘Walk’ is detected followed by the head posture ‘Head down’ within two seconds, the complex behavior network generates the atomic behavior ‘Sniffing’. In the latter network, the CEP engine receives and calculates the total frequency of the complex behavior-related atomic behaviors within an observation interval of 15 s. Then, the CEP system uses fuzzy CEP rules to detect whether the total frequency is abnormal, i.e., if the behavior happens more frequently when the owner is not at home [8,10,18]. Further details on the fuzzy function are presented in Section 2.4.3.

#### 2.4.3. Fuzzy Function of Dog Monitoring System

**Figure 5:**
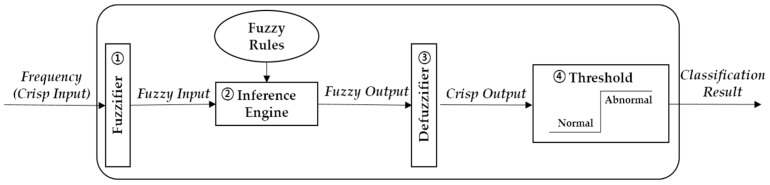
Fuzzy logic function structure of dog monitoring system.

**Figure 6:**
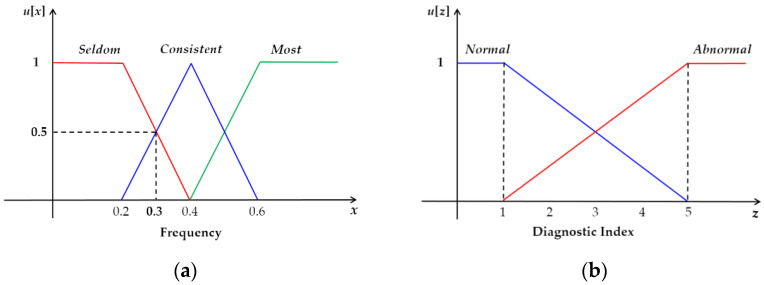
Membership Functions: (**a**) system input is complex behavior frequency (vocalization, exploratory and destructive) with fuzzy sets {Seldom(red), Consistent(blue), Most(green)}; (**b**) output is diagnosis index with fuzzy sets {Normal(blue), Abnormal(red)}.

Most of the dogs’ psychological separation anxiety knowledge is a natural language originating from experts [6,8,14,15,18,62]. Therefore, it is challenging to effectively express ambiguous and uncertain psychological knowledge using basic CEP rules. For instance, the symptomatic complex behavior ‘Excessive vocalization’ cannot be quantified to build pattern rules. To address this issue, we introduce fuzzy logic and expand the existing CEP rules in the complex behavior EPN. As a common approach to solving imprecise and vague problems, Fuzzy logic has a long history in automated clinical diagnosis [63,64]. Moreover, it is easier for experts to map their expertise into fuzzy logic than sophisticated probabilistic methods [40,41]. Figure 5 illustrates the fuzzy logic function structure of the proposed dog monitoring system, with the main steps described as follows:

1. Fuzzification: the fuzzifier applies the relevant membership functions to transform the crisp variables to fuzzy linguistic variables, whose values are natural language words instead of numerical values. This work utilizes domain knowledge [4,8,18,65], and thus the input linguistic variable is the frequency (f) of each complex behavior (destructive behavior, exploratory behavior, and vocalization). Specifically, F(f)={Seldom, Consistent, Most} is the set of decompositions for the linguistic variable frequency, with each *F(f)* member covering a portion of the overall frequency values. For example, in Figure 6a, the frequency is 30% (0.3) of the observation time, classified as 50% ‘Seldom’ and 50% ‘Consistent’. The fuzzifier transforms the crisp frequency input using the trapezoidal and triangular membership functions illustrated in Figure 6a. Similarly, the output linguistic variables are the symptom diagnosis indices involving two linguistic variables, i.e., {Normal, Abnormal}, with the trapezoidal membership function illustrated in Figure 6b.

2. Fuzzy rules and inference: based on the domain knowledge, the dog separation anxiety-detection fuzzy matrix is presented in Table 5. For instance, if the ‘Exploratory behavior’ is ‘Consistent’ or ‘Most’, the separation anxiety symptom state is ‘Abnormal’ [10].

3. Defuzzification: this stage utilizes the center of gravity [66], one of the most common defuzzifiers, to obtain the shape’s centroid generated by superimposing the fuzzy rules shapes.
4. Threshold Decision: based on the defuzzification result, a heuristic decision threshold is employed depending on the domain knowledge [4,18,65], which ultimately produces a final binary classification (normal or abnormal behavior). If the result exceeds a threshold, the complex behavior is diagnosed as the abnormal status ‘Excessive’ [67]. Further details on the fuzzy logic description can be found in [66,67,68].

The overall CEP-based dog behavior detection system is described in Algorithm 1. Specifically, the input event stream involves the posture events (*L*_1_) and the output of the separation anxiety-related atomic behaviors (*L*_2_) and complex behaviors (*L*_3_). Initially, the predefined rules in each event-processing network, linguistic variables, membership functions, and fuzzy logic rules are initialized (line 3). The overall system is defined by the event types: *L*_1_ posture, *L*_2_ atomic behavior, and *L*_3_ complex behavior events (lines 4–6). The upper case represents the event type, and the lower case represents the event instance. The fuzzy function is defined in lines 8–16. When the CEP system keeps receiving and creating events of different levels, the algorithm searches within the observation time for events satisfying the predefined rules of different level networks (lines 17–27). Once the events satisfy the rules, the CEP algorithm creates a behavior event and publishes the event to the event channel. Then, the system will return the detected Level-2 (*L*_2_) and Level-3 (*L*_3_) behaviors (line 28). For completeness, *p_i_*, *p*_*j*−1_, and *p_j_* are the postures to be detected in the atomic behavior network rules, and *L*_2*m*_ and *L*_2*k*_ denote the Level-2 stream, where each steam has the same type of atomic behaviors, with *m ≠ k*.
**Algorithm 1.** Complex Behavior Detection for SA.1**Input:***L*_1_ = {*p*_1_, *p*_2_, …, *p_i_*, …, *p_j_*, …, *p_n_*}2**Output:***L*_2_ = {*a*_1_, *a*_2_, …, *a_i_*, …, *a_j_*, …, *a_n_*}, *L*_3_ = {*c*_1_, *c*_2_,…, *c_i_*, …, *c_n_*}3**Initialize:** Pre-defined CEP rules, pre-defined linguistic variables, membership functions and fuzzy rules 4**Define** Posture event type = *P (id, s, posture, t)*5**Define** Atomic behavior event type = *A (id, atomic behavior, t_s_, t_e_)*6**Define** Complex behavior event type = *C (id, complex behavior, d, t_s_, t_e_)*7//Fuzzy function8**Function** F(frequent)9     Convert frequent to fuzzy values by membership functions10     Evaluate the rules in the rule base11     Combine the results of each rule12     results = Center of gravity calculation13     **If** results > Threshold14     **Then** classification result = Abnormal15     **Else** classification result = Normal16**Return** classification result17//Level 2 dog atomic behavior detection18**If** select * from *L*_1_19where repeat *p_i_. posture* more than two times ∧ *win (2 s)*20**Then** create *a_i_ (id, related atomic behavior, t_n_, t*_*n+*1_)21**If** select * from *L*_1_22where *p*_*j−*1_*. posture* → *p_j_. posture* ∧ *win (2 s)*23**Then** create *a_j_ (id, related atomic behavior, t_n_, t*_*n+*1_)24//Level 3 dog complex behavior detection25**If** select * from *L*_2_26where *F(C(L*_2*m*_*, L*_2*k*_*)) ∧*
*Win (15 s)*
*=*
*Normal or Abnormal*27**Then** create *c_i_ (id, related complex behavior, classification result of symptoms, t_s_, t_e_)*28**Return***L*_2_, *L*_3_

### 2.5. Dog Application Layer

In this layer, a web application is designed to report and analyze the monitoring results of separation anxiety-related complex behaviors in dogs. Upon automatically identifying the complex behaviors related to separation anxiety, the system sends the monitoring results to the owner or scientist.

## 3. Results

### 3.1. Data Collection and Datasets

The performance of the proposed detection monitoring system was evaluated using raw activity sensor data and video recordings of eight dogs recruited based on separation anxiety clinic criteria defined in [42]. Table 6 presents the basic information of the eight dogs.

**Figure 7:**
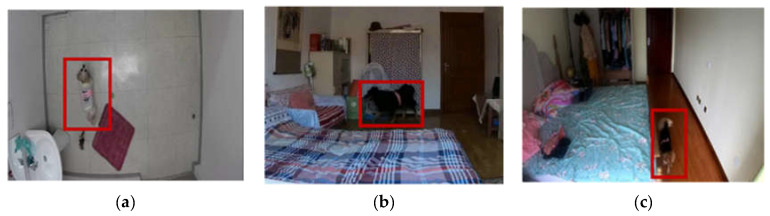
Examples of experimental areas for data collection. (**a**) Example of experiment conducted in laboratory; (**b**,**c**) examples of experiments conducted in owners’ apartments.

**Figure 8:**
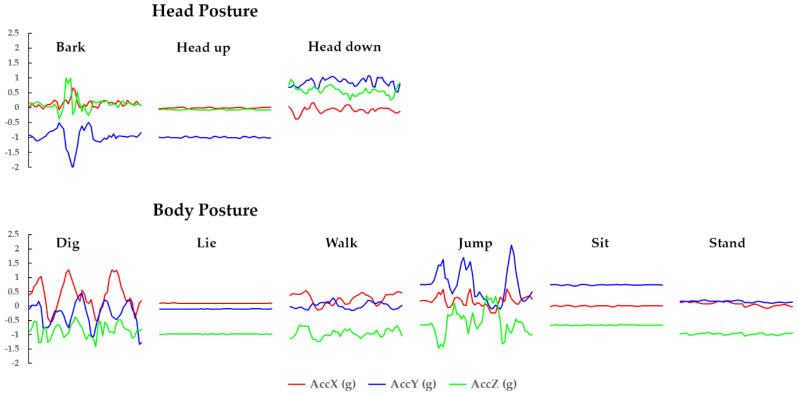
Visualized examples of each time-series data type (50 Hz).

The data were collected either in the owner’s premises or in the laboratory as shown in the examples in Figure 7. A red bounding box was used in Figure 7 to help identify the location of the dog in every example. The data collection procedure was conducted in three phases. (1) Preparation: mounting two lightweight motion sensors (LPMS-B2, size: 39 × 39 × 8 mm, weight: 12 g, sample rate: 50 Hz) on the dog’s neck and back to collect its head and body tri-axial accelerometer data. At this stage, a camera was set to record the dog’s activity with video and sound to use during the labeling and ensure the accuracy of the sensor data labels. (2) Synchronization: sensor synchronization by connecting the sensor to a computer via Low Energy Bluetooth. Sensor activation ensures uninterrupted video camera recording. (3) Activity recording: each dog is left to move for 5–15 min naturally and freely while its posture and movements are being recorded by the sensors and a video camera. The data received from the sensor contains six columns, namely sensor ID, frame number, timestamp, and three-axis accelerometer (*x*-axis, *y*-axis, *z*-axis). Figure 8 shows examples of raw time-series data of dog postures. The total duration of each activity is shown in Table 7. The dataset does not contain any missing values, and the data were chosen and classified (as ground truth) with the help of animal behavior researchers.

### 3.2. Implementation

**Figure 9:**
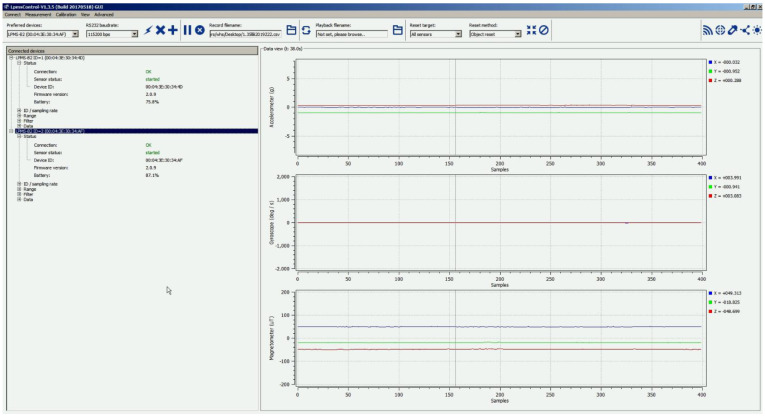
Screenshot of OpenMAT software used to capture tri-axial accelerometer signals.

The prototype system was implemented using a computer running an Intel i7 CPU with 64 GB RAM utilizing Windows 10 and a GTX 1080 Ti GPU. The system used OpenMAT software [69] to capture the tri-axial accelerometer signals. Figure 9 depicts a screenshot of OpenMAT software. The LSTM-based network was trained using TensorFlow library [70], and the CEP network was implemented using the Esper library as it provides a CEP engine and integrated tools for modeling CEP rules [71]. The web application based on the database was designed using Plotly and Dash Python libraries [72].

### 3.3. Evaluation

#### 3.3.1. Metrics

The recognition performance of each activity level is evaluated employing the Precision, Recall, and F1-score metrics [73]:

(4) $$\mathrm{Precision}=\frac{\mathrm{TP}}{\mathrm{TP}\;+\;\mathrm{FP}}$$

(5) $$\mathrm{Recall}=\frac{\mathrm{TP}}{\mathrm{TP}\;+\;\mathrm{FN}}$$

(6) $$F1\;\mathrm{score}=\frac{2\times \;\mathrm{Precision}\;\times \;\mathrm{Recall}}{\mathrm{Precision}\;+\;\mathrm{Recall}}$$

where true positive (TP) is the number of dog activities that are actually positive and classified as positive, False Positives (FP) is the number of dog activities that are actually negative and classified as positive, and False Negatives (FN) is the number of dog activities that are actually positive and classified as negative.

#### 3.3.2. Posture Monitoring Results (Level-1 Classification)

The first experiment was conducted to confirm the effectiveness of the stacked LSTM models proposed in this paper, and the second experiment to compare its performance with other models. The data used in the first and second experiments contained 4500 time-series samples. We made sure the data are balanced by using exactly 500 samples of data for each class. The division of training and testing data happens through the 5-fold cross-validation where a different fold containing 900 samples is used in every iteration. Hence, the data are divided for training and testing using a 8:2 ratio in every iteration, i.e., 3600 and 900 samples, respectively. Both stacked LSTM networks used for head and body posture recognition were trained using cross-entropy loss [46] and Adam Optimizer [74] with decay rates β_1_ of 0.9 and β_2_ of 0.999 and a learning rate of 0.0025. The batch size was 25, trained for 50 epochs.

The results of the first experiment, i.e., Level-1 head and body posture classification, using our proposed method obtained an average F1-score of 0.947 presented in Table 8, proving that it can accurately identify the dog’s head and body postures. Concerning the ‘Bark’ and ‘Head up’ postures, the accuracy of the model is relatively lower as some barks were not loud enough, which increased the difficulty to differentiate between the ‘Bark’ and ‘Head up’ postures. Additionally, high-intensity panting causes the dog’s head and body to move constantly, which adds noise to the signal. Similarly, some dog’s body postures such as ‘Stand’ or ‘Dig’ moved slightly, leading our model to predict ‘Walk’ or ‘Jump’ postures falsely. Moreover, some transactions between two postures exist during recognition, reducing the recognition effect.

In the second experiment, we compared the LSTM approach with two current dog activity classifiers [25,27,31], i.e., Naïve Bayes (NB) and Support Vector Machine (SVM). We employed the same training and testing datasets for all methods with five-fold cross-validation to guarantee a fair and accurate comparison, and statistical features (min, max, mean, standard deviation) were used to train the SVM and NB models. Table 9 shows the performance results using the F1-score and confirms that the stacked LSTM networks outperform current classifiers.

#### 3.3.3. Atomic Behavior Monitoring Results (Level-2 Classification)

Based on the results of Level-1 detection, we performed a Level-2 atomic behaviors identification experiment. This experiment considers 1070 dog atomic behavior data for Level-2 activity detection. Window slicing was used in this experiment for the data augmentation of some abnormal atomic behaviors that are exploited to detect the Level-3 behaviors [75,76]. Thus, the slicing window of 100-sample width and 50% overlap moved backward to augment ‘Escaping’ behaviors by 17 sequences and ‘Barking’ behaviors by 10 sequences. Then, an experiment was conducted to compare the recognition performance of Level-2 activities.

The first experimental results are presented in Table 10, revealing that the proposed system’s average detection accuracy approached 0.915. As summarized in Table 9, most dog atomic behaviors are correctly detected, confirming that the proposed method achieves good performance for Level-2 dog atomic behavior recognition.

In the second experiment, we compared our proposed method with SVM, Decision Tree (DT) and NB classifiers used in previous studies [24,25,27,31]. Similarly, in this experiment, the statistical features (min, max, mean and standard deviation) were used to train the SVM, DT, and NB models. As shown in Table 11, the proposed method (stacked LSTM + CEP) used the hierarchical structure achieved better performance results. SVM, DT and NB falsely recognized some of the ‘Sniffing’ behavior. This is because the activity is associated with head posture. It is relatively hard to distinguish the ‘Standing’ and ‘Sniffing’ only using a body sensor. Additionally, high-intensity panting ultimately increased atomic behaviors recognition error.

#### 3.3.4. Complex Behavior Monitoring Results (Level-3 Classification)

Two experiments were conducted to confirm the performance of Level-3 dog separation anxiety symptomatic complex behaviors detection. The first experiment used 152 destructive behavior samples, 84 vocalization samples, and 231 exploratory behavior samples. Fifty-six destructive behaviors were added through data augmentation using a slicing window of 750-sample width and 86.7% overlap. Likewise, 47 additional vocalization sequences and 122 exploratory behavior sequences were generated with data augmentation. The heuristic decision threshold for vocalization is 1.0, and the heuristic decision threshold for destructive and exploratory behaviors is 1.5. Similarly, this experiment used previous Level-2 activities experiment results as input data sent to the Fuzzy-CEP system to evaluate the performance of Level-3 complex behaviors detection.

Table 12 depicts the results of the first experiment, measuring the precision, recall, and F1-score metrics. The results revealed that our approach achieved an F1-score of 0.86 for symptomatic complex behaviors, highlighting that the hierarchical structure employed achieved an appealing performance. Based on the appealing performance, we conclude that the proposed CEP-based monitoring method is promising for detecting the dogs’ separation anxiety signs. The performance decline of Level-3 is primarily due to continuous false recognitions of the Level-1 to the Level-2 activities such as ‘Vocalization Behavior’. Hence, exploiting more sensors, e.g., a sound sensor, would further enhance our method’s performance.

The second experiment compared our proposed method with SVM, DT, and Random Forest (RF) classifiers for Level-3 complex behaviors. The experiment exploited several statistical features (min, max, mean, and standard deviation) to train the SVM, RF, and DT models. As shown in Table 13, the proposed method (stacked LSTM + Fuzzy-CEP) used the hierarchical structure combined with two sensors and achieved better performance results.

#### 3.3.5. Dog Monitoring System Web Application

**Figure 10:**
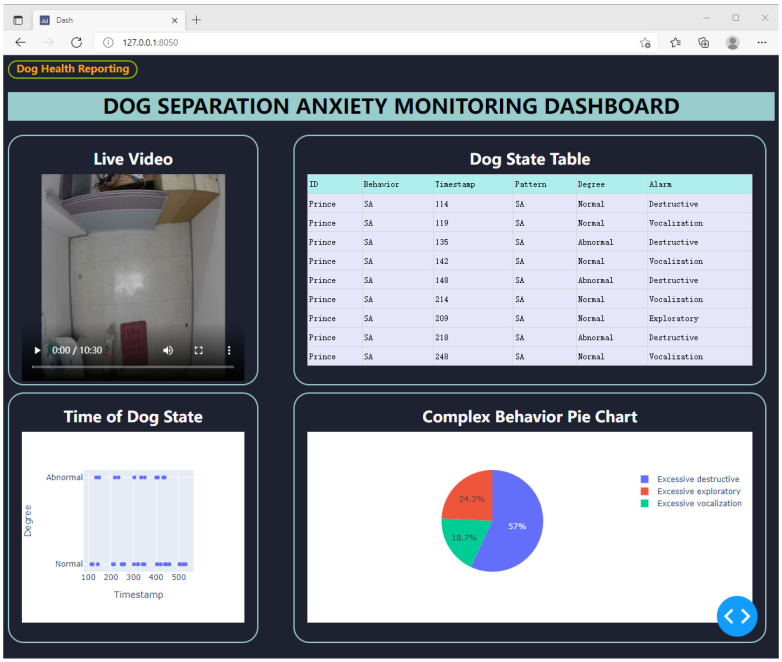
Web application of proposed dog monitoring system.

We designed a web application to report and analyze the detected dogs’ separation anxiety-related complex behavior. Figure 10 depicts a snapshot of our web application, including a live video stream to check the dogs’ activities, an alarm table, a time scatter chart showing the dog’s normal/abnormal status, and a pie chart analyzing the detected complex behaviors.

## 4. Conclusions

Owners leave their dogs at home alone, potentially causing psychological disorders such as separation anxiety, often accompanied by complex behavioral symptoms like excessive destructive behavior, excessive exploratory behavior, and excessive vocalization. In particular, those undesired complex behavioral symptoms are the main reason for the relinquishment of dogs. Thus, we present an appropriate monitoring method by developing a multi-level hierarchical system that automatically monitors freely moving home-alone dogs. The multi-level hierarchical system starts from Level-1 (fundamental head and body postures), goes through Level-2 (atomic behaviors), and reaches Level-3 (separation anxiety-related symptomatic complex behaviors). Regarding Level-1, we apply the stacked LSTM model to recognize the dog’s head and body postures using the time-series data extracted from wearable sensors. Then, based on the extracted postures, the CEP engine uses dog behavior knowledge-based pattern rules for Level-2 atomic behavior and Level-3 complex behavior identification. To overcome the limitations of basic CEP rules, this work proposes a Fuzzy-CEP, as fuzzy rules can handle the imprecision and vagueness represented through psychological knowledge. Our experiments evaluated the proposed approach using data collected from eight dogs recruited based on clinical inclusion criteria. The experimental results demonstrate that our system achieves approximately an F1-score of 0.86, affording an appealing dog symptomatic complex behavior monitoring scheme appropriate for a real-world environment. Furthermore, the experiments reveal that our approach can provide a feasible way to describe complex behaviors related to psychiatric symptoms and help promote the implementation of artificial intelligence technology in the veterinary field. Our subsequent study intends to develop a robust dog behavior monitoring system to monitor separation anxiety symptoms by combining sensor, video, and sound data.

## Figures and Tables

**Table 1:** Dog monitoring system hierarchy and activity definitions. (Types: (M)—Motion; (P)—Pose).

Level	Category	Name (Type)	Description	Related Lower-Level Activity	Observation Time
Level 1	Head posture	Up (P)	Head is higher than the shoulders and body.	-	1 s
		Down (P)	Head is lower than shoulders and body.	-	
		Bark (M)	Bark movement.	-	
	Body posture	Walk (M)	Gait motion.	-	
		Lie (P)	Side of the dog is in contact with the ground.	-	
		Sit (P)	Haunches are on the ground, and elbows are not in contact with the environment.	-	
		Stand (P)	All feet are on the ground without moving.	-	
		Dig (M)	Forelegs consecutively or concurrently move with each other.	-	
		Jump (M)	Both of the dog’s forelegs or all legs leave the ground.	-	
Level 2	Atomic behavior	Sniffing	Head downwards and close to the floor, while the dog is walking or standing.	Walk, Stand, Head down	2 s
		Escaping	Repetitive jumps represent an attempt of escape.	Jump	
		Barking	Repetitive barks.	Bark	
		Walking	Walk for more than 1 s.	Walk	
		Lying	Lie for more than 1 s.	Lie	
		Sitting	Sit for more than 1 s.	Sit	
		Standing	Stand for more than 1 s.	Stand	
		Digging	Dig for more than 1 s.	Dig	
Level 3	Symptomatic complex behavior	Excessive destructive behavior	The dog is digging at a high frequency, possibly attempting to escape from exit points.	Escaping, Digging	15 s
		Excessive exploratory behavior	The dog is walking around in the house, sniffing at different objects, and nosing at and around the door, with a high frequency.	Walking, Sniffing	
		Excessive vocalization	The dog is repetitively barking, howling, or whining for a long time.	Multiple barking	

**Table 2:** Recent automatic recognition research of dog activities (published between 2009–2021). (Types: (D)—Disease-related behavior).

Level	Sensors	Location	Technique	Target	Ref.
1	Accelerometer	Back	Pose Estimation algorithm	Body posture	[19]
	Camera	Ceiling	Semisupervised approach	Body posture	[20]
	Accelerometer	Neck, back	Knowledge engineering approach	Body posture	[21]
	Gyroscope	Neck	Rule-based approach	Head posture	[22]
2	Accelerometer	Neck	Neural Networks (NN), Instance-based learning (IBk), Random Forest (RF)	Atomic behavior	[23]
	Accelerometer, gyroscope	Body	Decision Tree (DT), Hidden Markov Model (HMM)	Atomic behavior	[24]
	Accelerometer, gyroscope	Back	Support Vector Machine (SVM)	Atomic behavior	[25]
	Accelerometer, gyroscope	Neck	Not specified	Atomic behavior	[26]
	Camera, accelerometer, angular velocity	Neck, back, thigh, waist	SVM	Atomic behavior	[27]
	Accelerometer	Neck	Linear and quadratic discriminant analysis	Atomic behavior	[28]
	Accelerometer	Neck	K-Nearest Neighbor (KNN)	Atomic behavior (D)	[17]
	Accelerometer	Neck	Dynamic Time Warping (DTW)	Atomic behavior (D)	[29]
	Accelerometer	Neck	Rule-based bio-inspired approach	Pruritic behavior (D)	[30]
	Accelerometer, gyroscope	Neck, tail	Artificial Neural Network (ANN), Naïve Bayes (NB), RF, SVM, KNN	Atomic behavior and emotion	[31]
	Microphone, camera	Not specified	Convolutional Neural Network (CNN)	Reducing separation anxiety (D)	[32]
	Accelerometer	Neck	Machine learning (Not specified)	Atomic behavior (D)	[33]

**Table 3:** Event constructors for dog behavior detection.

Constructor	Symbol	Expression	Meaning
And	∧	*E*_1_ ∧ *E*_2_	Conjunction of events *E*_1_ and *E*_2_
Or	∨	*E*_1_ ∨ *E*_2_	Disjunction of events *E*_1_ and *E*_2_
Repeat	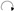	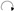 *E*_1_	Repeat of *E*_1_ events
Follow	→	*E*_1_→*E*_2_	*E*_1_ occurs followed by *E*_2_
Count	*C( )*	*C(E*_1_ *)*	Calculation of the frequency of *E*_1_
Window	*Win()*	*Win(t)*	Observation time interval *t*
Fuzzy	*F( )*	*F(E*_1_ *)*	Fuzzy logic calculation of *E*_1_

**Table 4:** Event processing pattern rules expression for dog behavior detection.

EPN	Rule Type	CEP Rules Definition	Example
Atomic Behavior EPN	C_1_	In two-second observation time interval, the state maintains the same postures *P* without any change.	Digging: 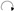 *Dig* ∧ *Win(2 s)*
	C_2_	In two-second observation time interval, *P*_1_ occurs followed by *P*_2_.	Sniffing: *(Walk*→*Head down)* ∧ *Win (2 s)*
Complex Behavior EPN	A	In 15-s observation time interval, count the total frequency of *a*_1_ and *a*_2_ occurrences and calculate the fuzzy function result.	Excessive Exploratory: *F(C (Walking* ∨ *Sniffing)* ∧ *Win (15 s))* = *Abnormal*

**Table 5:** Fuzzy matrix for dog psychological separation anxiety symptoms monitoring.

Diagnosis Index	Seldom	Consistent	Most
Destructive Behavior	Normal	Abnormal	Abnormal
Exploratory Behavior	Normal	Abnormal	Abnormal
Vocalization	Normal	Abnormal	Abnormal

**Table 6:** Basic information of subject dogs.

Serial	Size	Name	Breed	Age
1	Small	Kimi	Maltese	0.5
2	Small	Prince	Papillon	9
3	Small	Doudou	Mix	1
4	Small	Tufei	Mix	1.5
5	Small	Lili	Papillon	7
6	Medium	Coco	Mix	4
7	Medium	Puding	Mix	0.5
8	Large	Coffee	Mix	7

**Table 7:** Total duration of each activity.

Level	Category		Total Duration
Level 1	Head posture	Bark	10.7 min
		Head down	18.4 min
		Head up	33.8 min
	Body posture	Dig	13.3 min
		Jump	11.5 min
		Lay	12.0 min
		Sit	11.0 min
		Stand	18.9 min
		Walk	20.4 min
Level 2	Atomic behavior	Sniffing	10.0 min
		Escaping	8.5 min
		Barking	8.4 min
		Walking	12.3 min
		Lying	8 min
		Sitting	6.8 min
		Standing	12.3 min
		Digging	9.3 min
Level 3	Symptomatic complex behavior	Destructive behavior	48.5 min
		Exploratory behavior	72.3 min
		Vocalization	25 min

**Table 8:** Overall precision, recall, and F1-score of Level-1 postures.

Level		Two-Layer Stacked LSTM			
	Category		Precision	Recall	F1-Score
Level 1	Head posture	Bark	0.944	0.904	0.922
		Head down	0.996	0.998	0.997
		Head up	0.914	0.946	0.929
	Body posture	Dig	0.894	0.889	0.889
		Jump	0.879	0.878	0.876
		Lie	0.990	0.991	0.990
		Sit	0.988	0.994	0.992
		Stand	0.963	0.967	0.975
		Walk	0.962	0.947	0.954
	Average		0.948	0.946	0.947

**Table 9:** Comparison of Level-1 posture identification performance.

Category		F1-Score		
		Proposed Method	SVM	NB
Head posture	Bark	**0.922**	0.856	0.665
	Head down	**0.997**	0.853	0.719
	Head up	0.929	**0.990**	0.978
Body posture	Dig	0.889	**0.969**	0.935
	Jump	0.876	**0.950**	0.919
	Lie	0.990	**0.996**	0.996
	Sit	**0.992**	0.678	0.644
	Stand	**0.975**	0.746	0.674
	Walk	0.954	**0.976**	0.970
Average		**0.947**	0.890	0.833

**Table 10:** Overall precision, recall, and F1-score of Level-2 atomic behaviors.

Level	Stacked LSTM + CEP				
	Category	Num.	Precision	Recall	F1-Score
Level 2	Sniffing	152	0.909	0.921	0.915
	Escaping	105	0.920	0.981	0.949
	Barking	101	0.876	0.842	0.859
	Walking	220	0.980	0.891	0.933
	Lying	90	0.987	0.844	0.910
	Sitting	55	0.981	0.946	0.963
	Standing	218	0.906	0.844	0.874
	Digging	129	1.000	0.822	0.902
	Average		0.945	0.886	0.915

**Table 11:** Comparison of Level-2 atomic behaviors identification performance.

Category	F1-Score			
	Proposed Method	SVM	DT	NB
Sniffing	**0.915**	0.794	0.869	0.757
Escaping	**0.949**	0.824	0.821	0.667
Barking	**0.859**	0.833	0.745	0.672
Walking	0.933	**0.951**	0.948	0.914
Lying	0.910	0.909	**0.953**	0.931
Sitting	**0.963**	0.672	0.931	0.657
Standing	0.874	0.721	**0.926**	0.564
Digging	0.902	0.907	0.917	**0.919**
Average	**0.915**	0.827	0.889	0.760

**Table 12:** Overall precision, recall, and F1-score of Level-3 complex behaviors.

Level	Stacked LSTM + Fuzzy-CEP					
	Category		Num.	Precision	Recall	F1-Score
Level 3	Destructive Behavior	Abnormal	91	0.888	0.868	0.878
		Normal	61	0.810	0.836	0.823
	Exploratory Behavior	Abnormal	168	0.940	0.929	0.934
		Normal	63	0.815	0.841	0.828
	Vocalization Behavior	Abnormal	54	0.891	0.907	0.899
		Normal	30	0.828	0.800	0.814
	Average			0.862	0.864	0.863

**Table 13:** Comparison of Level-3 complex behaviors identification performance.

Level	Category		F1-Score			
			Proposed Method	SVM	DT	RF
Level-3	Destructive Behavior	Abnormal	0.878	0.859	0.878	**0.882**
		Normal	**0.823**	0.736	0.748	0.760
	Exploratory Behavior	Abnormal	**0.934**	0.706	0.630	0.561
		Normal	**0.828**	0.523	0.537	0.500
	Vocalization Behavior	Abnormal	**0.899**	0.493	0.667	0.608
		Normal	**0.814**	0.611	0.690	0.652
	Average		**0.863**	0.655	0.692	0.660
